# Aortic Thrombus Extending to Left Subclavian in a Patient With Diffuse Venous Thromboembolism on Aromatase Inhibitor Therapy

**DOI:** 10.7759/cureus.16698

**Published:** 2021-07-28

**Authors:** Mrhaf Alsamman, Joshua Pothen, Marialla Inoyatov, Elis M Cruz Salcedo, Chembu Ramesh

**Affiliations:** 1 Internal Medicine, Health Corporation of America-University of Central Florida (HCA-UCF) Consortium, Ocala, USA; 2 Anesthesia, Health Corporation of America-University of Central Florida (HCA-UCF) Consortium, Ocala, USA; 3 Internal Medicine, Ocala Regional Medical Center, Ocala, USA

**Keywords:** venous thromboembolism (vte), thrombus formation, acute pulmonary embolism, deep vein thrombosis (dvt), subclavian occlusion

## Abstract

Concomitant arterial and venous thrombosis is an infrequent event often associated with malignancy, hyperhomocysteinemia, and thrombophilic conditions. Some overlapping pathophysiology mechanisms suggest an association between arterial and venous thrombosis. It is reported that thrombosis in the arterial and venous systems develops through distinct mechanisms affecting inflammatory and oxidative pathways. Recently, the aromatase inhibitors have moved to the forefront of adjuvant hormonal therapy, however, the adverse effects of these agents are not yet fully understood. It is generally accepted that tamoxifen, but not aromatase inhibitors, is associated with an increased risk of thrombosis in women with breast cancer. Here, we report an unusual case of an 87-year-old female on anastrozole therapy with aortic thrombus extending into the left subclavian artery with associated diffuse venous thromboembolism (VTE).

An 87-year-old-female with a history of breast cancer in remission, obesity, hypertension, and dyslipidemia presented to the emergency department with new onset of left arm weakness and tingling sensation. Vital signs showed respiratory rate of 20 per minute, oxygen saturation of 95% on 3 L of oxygen via nasal cannula, blood pressure of 150/79 mmHg, and pulse 81 beats per minute. Computed tomography angiography (CTA) neck showed an aortic thrombus extending into the left subclavian artery and bilateral pulmonary emboli (PE). Doppler ultrasound of the lower extremities showed a deep venous thrombosis (DVT) in the left lower extremity. Echocardiography showed no patent foramen ovale. She was started on continuous heparin infusion and subsequently transitioned to an oral anticoagulation medication upon discharge.

Symptomatic ischemic lesions of the upper extremity due to thrombosis of the subclavian artery are extremely rare, occurring in less than one percent of the population. While this patient had a history of early-stage breast cancer, she was on adjuvant anastrozole therapy with no evidence of recurrence or further tumor burden as per her outpatient oncologist, who also followed her during her hospital stay. She also had no prior history of thromboembolic disease or clotting disorders. Her only risk factors appear to be her age and her obesity (with a BMI over 30). Nevertheless, the extent of thromboembolism seen in this patient is greater than that might be expected with these factors.

This case highlights a concomitant rarity of arterial and venous thrombosis. Also, there are not enough studies on anastrozole effect on thromboembolism. Given these risk factors, we recommend a high degree of suspicion for VTE in patients who are on anastrozole therapy.

## Introduction

Concomitant arterial and venous thrombosis is an infrequent event and is mostly associated with malignancy, hyperhomocysteinemia, and thrombophilic conditions [1,2]. Several theories have proposed age to be a contributor of arterial and venous thrombosis via mechanism that include structural or functional change in the vascular wall and increased systemic activation of blood coagulation potentially due to reduced exercise levels and increased periods of immobility [3]. Thrombophilic conditions have strong risk factors for venous thromboembolisms (VTE); however, their effect on arterial thrombosis is less understood, except in the case of antiphospholipid antibody syndrome which is associated with both arterial and venous thrombosis and complications during pregnancy [3]. Association between malignancy and VTE is well established; however, arterial thrombosis has more recently being recognized as a complication and possible independent risk factor of cancer with an estimated incidence of 1.5-5.2% among episodes of myocardial infarction, cerebrovascular events, and peripheral artery disease [4]. Studies comparing aromatase inhibitors to tamoxifen in the adjuvant setting have reported a lower rate of venous thromboembolism with the aromatase inhibitors, yet the incidence of venous thromboembolism with these new agents is higher than that expected in the general population. Here, we report an unusual case of an 87-year-old female on anastrozole therapy with aortic thrombus extending into the left subclavian artery along with diffuse VTE.

## Case presentation

**Figure 1:**
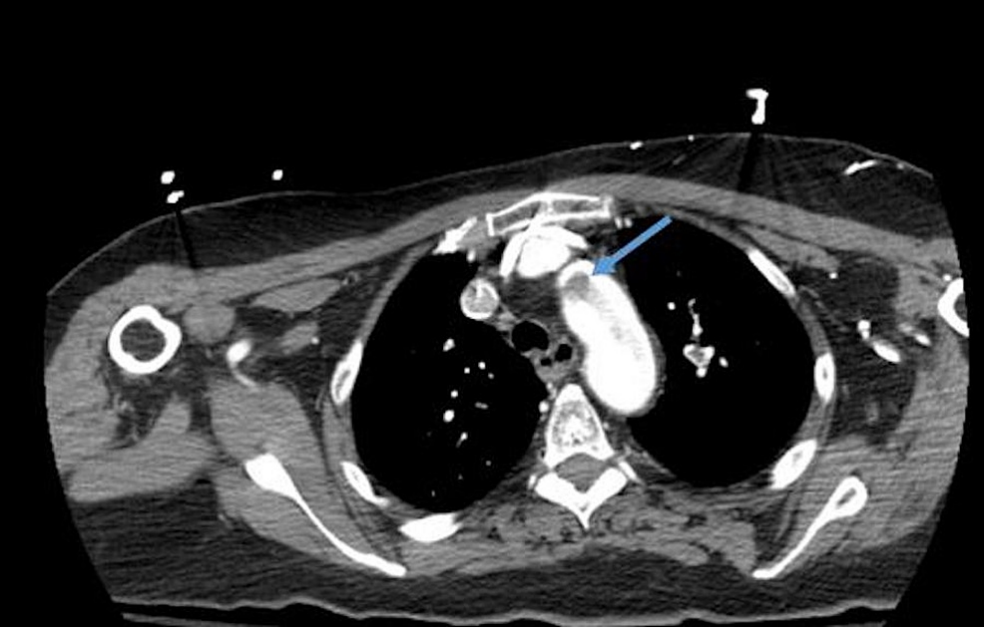
Computed tomography angiography (CTA) - blue arrow showing thrombus in aortic arch

**Figure 2:**
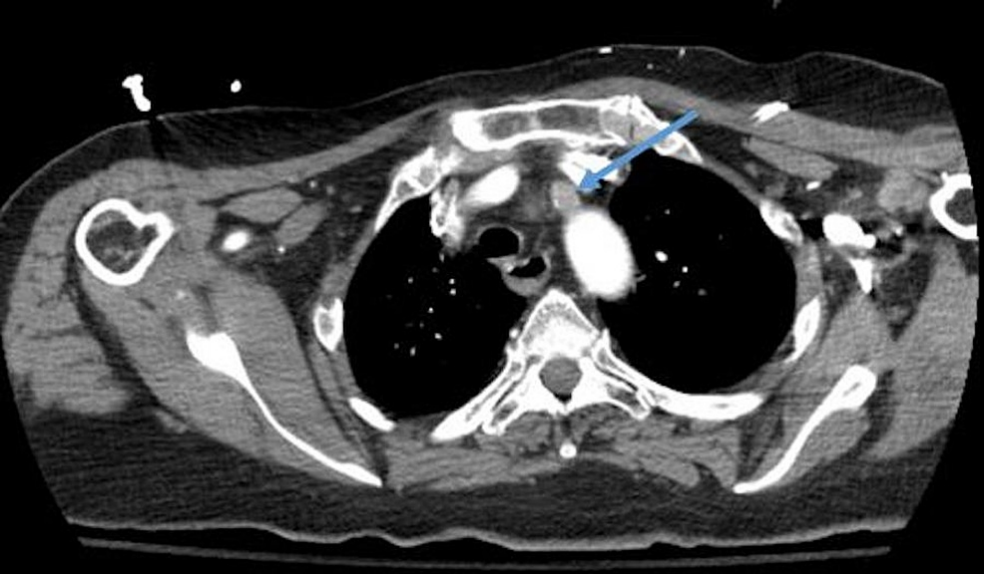
Computed tomography angiography (CTA) - blue arrow showing thrombus extending to left subclavian artery

**Figure 3:**
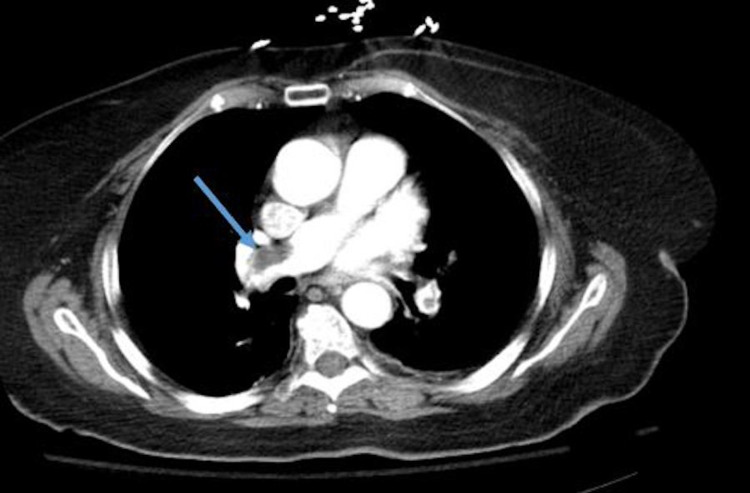
Computed tomography angiography (CTA) - blue arrow showing right pulmonary embolism

**Figure 4:**
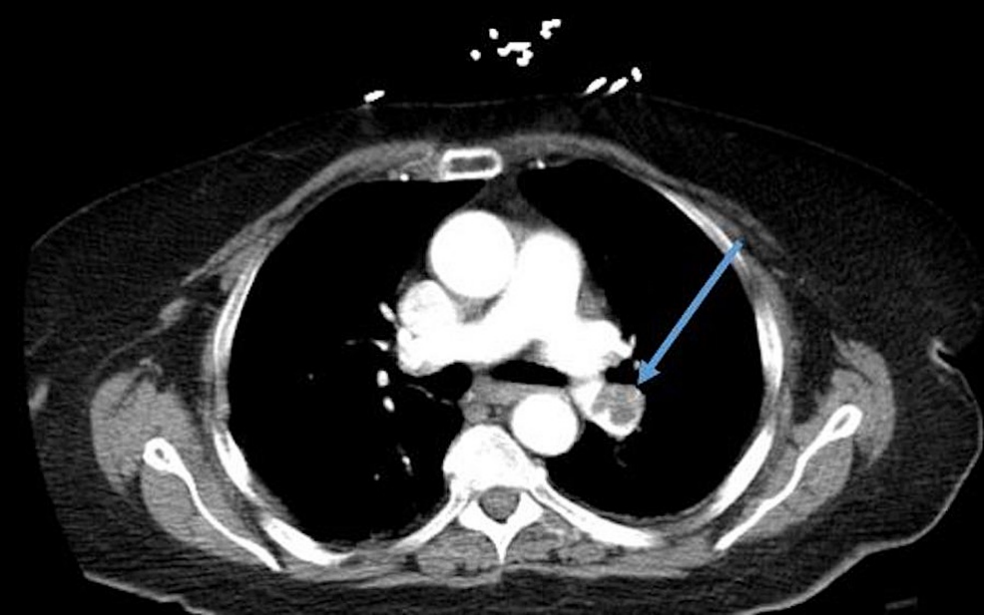
Computed tomography angiography (CTA) - blue arrow showing left pulmonary embolism

An 87-year-old female with a history of breast cancer diagnosed in 2006, treated with lumpectomy and in remission since then, obesity, well-controlled hypertension, and dyslipidemia presented to the emergency department with new onset of left arm weakness and tingling sensation. The patient was sitting in her chair when her caregiver noticed weakness in her left upper extremity. The patient was stroke alerted on admission. Upon admission, the patient had a National Institutes of Health Stroke Scale (NIH) of 2. Vital signs showed respiratory rate of 20 per minute, oxygen saturation of 95% on 3 L of oxygen via nasal cannula, blood pressure of 150/79 mmHg, and pulse 81 beats per minute. CT brain did not show any signs of stroke. The patient was not given tissue plasminogen activator (tPA) due to low NIH score. However, CTA neck showed aortic thrombus extending into the left subclavian artery (Figures 1, 2) and bilateral pulmonary emboli (Figures 3, 4). CTA neck also showed a large nodule in the left thyroid lobe measuring approximately 6.4 cm in size. Doppler ultrasound of the lower extremities showed DVT in the left lower extremity. The patient was subsequently started on continuous heparin Infusion. Echocardiography showed estimated ejection fraction of 60% with no patent foramen ovale. Patient was evaluated by vascular surgery and recommended no acute intervention as left upper extremity has adequate perfusion. Thyroid mass was biopsied and showed mostly inflammatory cells, blood elements, and rare follicular cells. However, previous records showed a known non-malignant adenoma. Left upper extremity weakness has significantly improved. The patient was transitioned to and oral anticoagulation medication upon discharge.

## Discussion

Our patient represents an unusual case regarding the mild symptoms occurred in the setting of widespread thrombosis. Not only the patient had left subclavian artery thrombosis due to an extensive aortic thrombosis, but the patient also had a left lower extremity deep vein thrombosis and bilateral pulmonary embolism. Interestingly, the left arm weakness was the only symptom reported by the patient, with no other symptoms such as other extremity pain or shortness of breath.

While this patient had a history of early-stage breast cancer diagnosed in 2006 and treated with lumpectomy, she was on adjuvant anastrozole therapy and had no evidence of recurrence or further tumor burden as per her outpatient oncology team, who also followed her during her hospital stay. She also had no prior history of thromboembolic disease or clotting disorders. In fact, her only risk factors appear to be her age and her obesity (with a BMI over 30). Nevertheless, the extent of thromboembolism seen in this patient is greater than might be expected. The most mature data on VTE incidence in women treated with aromatase inhibitors comes from the Arimidex, Tamoxifen, Alone, or in Combination (ATAC) study. This study enrolled >9000 women, who were randomized to tamoxifen alone, anastrozole alone, or the combination of the two drugs. The combination arm was stopped when no significant difference was found between the tamoxifen alone arm and the combination arm. The incidence of VTE at four years of follow-up was higher in the tamoxifen arm (RR=1.7 compared to anastrozole), but importantly the incidence in the anastrozole alone arm was 1.1%, which corresponds to an absolute risk of approximately 3.6 per 1000 women per year. This is greater than the estimated risk in untreated healthy women [4].

The effects of aromatase inhibitors (AI) on the coagulation system are not well-studied. The potential mechanism of aromatase inhibitors contributing to an increased risk of VTE is not clear, as it has been shown that aromatase inhibitors decrease estrogen levels beyond already low postmenopausal levels. To our knowledge, there is only one published study examining the effects of an AI on the coagulation system [5]. In this study, the second-generation AI, fadrozole hydrochloride, which is not approved in the United States, did not cause any significant changes in coagulation factors, including ATIII, protein C, and protein S, with the exception of a significant increase in the acute phase reactant fibrinogen, in 21 postmenopausal women with advanced breast carcinoma. It is possible that the aromatase inhibitors could contribute to thrombotic risk by an inflammatory mechanism not directly mediated by estrogen [4]. Anticoagulation remains the cornerstone of treatment for concomitant arterial and venous thromboembolic disease. In recent years, direct oral anticoagulants (DOACs) now preferred over vitamin K antagonist therapy due to the relative ease of administration. However, the latter is preferred if both arterial and venous thromboembolism occur in the setting of antiphospholipid syndrome. Some surgical options to remove subclavian thrombus include axillary-axillary bypass, carotid-subclavian bypass, and transposition of the subclavian artery. Percutaneous transluminal angioplasty with stenting is currently the best modality for relieving the thrombus. Contraindications for surgical intervention include inadequate distal runoff, inadequate vessel size, enhanced collateral circulation of the occluded area, and medical health contraindications [6].

Our patient was initially managed on a continuous heparin infusion due to concern for limb ischemia. However, as the patient remained vitally stable and found to have good limb perfusion, she did not require surgical or thrombolytic therapy. Patient was safely discharged on apixaban therapy.

## Conclusions

This case highlights a concomitant rarity of arterial and venous thrombosis. There are not enough studies on anastrozole effect on thromboembolism. Given these risk factors, we recommend a high degree of suspicion for VTE in patients who are on anastrozole therapy.

## References

[1] Kutiyal AS, Dharmshaktu P, Kataria B, Garg A (2016). A rare occurrence of simultaneous venous and arterial thromboembolic events - lower limb deep venous thrombosis and pulmonary thromboembolism as initial presentation in acute promyelocytic leukemia. Clin Med Insights Oncol.

[2] Lowe GD (2008). Common risk factors for both arterial and venous thrombosis. Br J Haematol.

[3] Previtali E, Bucciarelli P, Passamonti SM, Martinelli I (2011). Risk factors for venous and arterial thrombosis. Blood Transfus.

[4] Lycette JL, Luoh SW, Beer TM, Deloughery TG (2006). Acute bilateral pulmonary emboli occurring while on adjuvant aromatase inhibitor therapy with anastrozole: case report and review of the literature. Breast Cancer Res Treat.

[5] Costa LA, Kopreski MS, Demers LM, Chinchilli VM, Santen RJ, Harvey HA, Lipton A (1999). Effect of the potent aromatase inhibitor fadrozole hydrochloride (CGS 16949A) in postmenopausal women with breast carcinoma. Cancer.

[6] Hassan SA, Akhtar A, Falah NU, Khan M, Zahra U (2020). A case of subclavian artery thrombosis. Cureus.

